# Maternal Thyroid Autoantibodies: Colborn’s Response

**Published:** 2004-11

**Authors:** Theo Colborn

**Affiliations:** The Endocrine Disruption Exchange, Paonia, Colorado, E-mail: colborn@tds.net

I thank Koppe for raising the question of the significance of the presence of increased thyroid peroxidase antibodies (TPO-Ab) during neurodevelopment or even later in life. I have wondered for years why medical practitioners and laboratories do not routinely quantify TPO-Ab in blood screening for thyroid disorders. High priority should be given to learning more about the relationship between the combination of high TPO-Ab and low free thyroxine (fT_4_), and impaired IQ and psychomotor development and the possible role of foreign substances such as polychlorinated biphenyls (PCBs) in these changes. Although the value of routine antithyroglobin antibody (TG-Ab) testing is being questioned, in future epidemiologic studies looking at the role of PCBs in neurodevelopment perhaps TG-Ab should be included in the design as well. It might prove enlightening to also routinely test for TG-Ab at several research/medical institutions to continue to explore this immune connection with the thyroid economy. Also, perhaps it is time to explore the nutritional state (protein consumption, quality and quantity of serum proteins) of the mother and her unborn child during gestation, which might contribute to the conflicting findings among the various cohort studies about the role of PCBs in neurodevelopment. In the meantime, until more is understood about neurodevelopmental impairment, I would like to take this opportunity to reinforce the need to routinely test all pregnant women and those planning to become pregnant for fT_4_, free triiodothyronine, thyroid-stimulating hormone, and TPO-Ab. Information such as this would allow for intervention, if needed, to prevent irreversible brain damage.

